# Semiclassical Modified Redfield and Generalized Förster Theories of Exciton Relaxation/Transfer in Light-Harvesting Complexes: The Quest for the Principle of Detailed Balance

**DOI:** 10.1021/acs.jpcb.1c01479

**Published:** 2021-06-14

**Authors:** Thomas Renger

**Affiliations:** Institut für Theoretische Physik, Johannes Kepler Universität Linz, Altenberger Str. 69, 4040 Linz, Austria

## Abstract

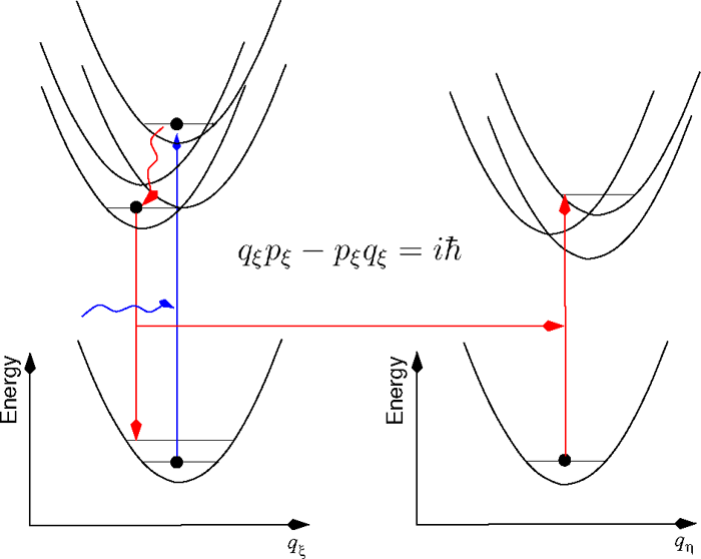

A conceptual problem of transfer theories that use a semiclassical description of the electron-vibrational coupling is the neglect of the correlation between momenta and coordinates of nuclei. In the Redfield theory of exciton relaxation, this neglect leads to a violation of the principle of detailed balance; equal “uphill” and “downhill” transfer rate constants are obtained. Here, we investigate how this result depends on nuclear reorganization effects, neglected in Redfield but taken into account in the modified Redfield theory. These reorganization effects, resulting from a partial localization of excited states, are found to promote a preferential “downhill” relaxation of excitation energy. However, for realistic spectral densities of light-harvesting antennae in photosynthesis, the reorganization effects are too small to compensate for the missing coordinate–momentum uncertainty. For weaker excitonic couplings as they occur between domains of strongly coupled pigments, we find the principle of detailed balance to be fulfilled in a semiclassical variant of the generalized Förster theory. A qualitatively correct description of the transfer is obtained with this theory at a significantly lower computational cost as with the quantum generalized Förster theory. Larger deviations between the two theories are expected for large energy gaps as they occur in complexes with chemically different pigments.

## Introduction

The high efficiency of photosynthetic light harvesting relies on a directed energy transfer to the photosynthetic reaction center, where the excitation energy is trapped by primary electron transfer reactions.^1−4^ The directionality is achieved by tuning the excited states of the light-harvesting complexes such that the low-energy states are located close to the reaction center. These effects are achieved by exploiting the pigment–pigment as well as the pigment–protein coupling.^5,6^

In the photosynthetic apparatus of purple bacteria,^7−10^ the number of strongly coupled bacteriochlorophyll *a* (BChl *a*) pigments is increased between the peripheral LH2 and the core LH1 light-harvesting complex surrounding the reaction center (RC). Due to the Coulomb coupling between electrons of different pigments, their motion becomes correlated, and delocalized excited states (exciton states) are formed with shifted excitation energies and redistributed oscillator strengths as compared to isolated or weakly coupled pigments (also present in LH2 but not in LH1). The larger number of strongly coupled pigments in LH1^11−13^ than in LH2^14−16^ contributes to the fact that the low-energy exciton states of LH1 are red-shifted with respect to those of LH2, providing a driving force for directed energy transfer. Finally, the excitation energy is trapped by the special pair, a BChl *a* dimer in the reaction center. In this case, nature exploits electron exchange in the special pair,^17−19^ in addition to the Coulomb coupling, to reach a low enough excitation energy in order to accept the excitation energy from the low-energy exciton states of LH1.

In green sulfur bacteria, an outer antenna system (the chlorosome) is connected to the reaction center complex via the baseplate and the Fenna–Matthews–Olson (FMO) protein.^20,21^ The local excitation energies (site energies) of the BChl *a* pigments in the FMO protein are tuned by electrostatic pigment–protein coupling^22−24^ such that a site energy funnel toward the reaction center complex is created.^25−27^ Due to the different site energies of the pigments, the exciton states in the FMO protein are partially localized.^25,28^ The low-energy exciton states face the reaction center complex,^21,25,26^ and hence, the relaxation between high- and low-energy exciton states leads to a spatially directed energy transfer.

The fundamental principle behind the preferential “downhill” transfer of excitation energy is the detailed balance of rate constants
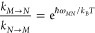
1for transfer between two states |*M*⟩ and |*N*⟩ that are separated by an energy difference *ℏω*_*MN*_, where *k*_B_ is Boltzmann’s constant, and *T* is the temperature.

The detection of long-lived oscillating signals in 2D electronic spectroscopy on the FMO protein^29,30^ and marine cryptophyte algae^31^ triggered fundamental questions on the role of quantum effects in photosynthesis and other biological and chemical systems.^28,32−34^ It is understood now that there is a close analogy between delocalized excited electronic states and classical coupled electronic harmonic oscillators.^35−40^ However, an important quantum aspect concerns the relaxation of excitons. For weak exciton-vibrational coupling, a classical treatment of nuclear motion leads to a violation of the principle of detailed balance.^28,39^ Equal “uphill” and “downhill” relaxation rate constants are obtained. This defect of the classical theory can be traced back to the absent correlation between classical momenta and coordinates that arise in a quantum theory from the uncertainty principle.

In the case of strong exciton-vibrational coupling, where nuclear reorganization effects accompany the energy transfer, the importance of nuclear quantum effects is less clear. A recent study by Reppert and Brumer,^40^ treating the electronic as well as nuclear degrees of freedom classically, finds that the nuclear reorganization effects are suppressed, and the principle of detailed balance is still violated such that the “uphill” is equal to the “downhill” rate constant, as for weak exciton-vibrational coupling discussed above. This result contrasts semiclassical Marcus theory of nonadiabatic electron transfer,^41,42^ which strictly fulfills the principle of detailed balance, despite a classical treatment of nuclear motion. In a semiclassical study by Ishizaki and Fleming,^43^ exciton dynamics was investigated in the basis of localized excited states, and nuclear motion was treated classically, neglecting nuclear reorganization effects. The authors justified this neglect by an unphysical Ehrenfest force in the equations of motion for the nuclear coordinates for intermediate occupation probabilities of localized excited states (in the case of exciton delocalization) and suggested that a treatment with Tully’s surface hopping approach^44−46^ might lead to the correct equilibrium population of excited states. There is indeed a long history of methods^44,47−50^ that try to find a simple and yet accurate quasiclassical description of quantum behavior including the detailed balance condition.^45,46,48,51^

In the present work, rather than investigating ways to mimic the quantum behavior, we introduce potential energy surfaces (PESs) of delocalized excited states and derive expressions for rate constants for transitions to different PESs assuming either a classical or a quantum nuclear motion in the PES of the initial exciton state. An important point of our model is that the mutual displacements of excitonic PESs along the coordinate axes are taken into account as well as their displacement with respect to the PES of the electronic ground state. In this way nuclear reorganization effects are included. We investigate exciton relaxation in domains of strongly coupled pigments and between such domains with weak interdomain couplings, leading to the semiclassical modified Redfield theory and semiclassical generalized Förster theory, respectively.

The theories are applied to the intramonomer exciton relaxation and the intermonomer excitation energy transfer in the trimeric FMO protein of green sulfur bacteria, with a particular focus on the detailed balance condition and the comparison of the quantum and semiclassical transfer kinetics.

## Theory

### Hamiltonian

We use a standard Frenkel exciton Hamiltonian expanded in the basis of localized electronic states , where chromophore *m* is in the electronic excited state, and all other chromophores *k* ≠ *m* are in their electronic ground state; *r* comprises the electronic coordinates of the chromophores. The wave function overlap between different chromophores is assumed to be sufficiently small such that there is no electron exchange between them. The Hamiltonian of singly excited states reads^42,52,53^
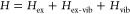
2where the exciton part
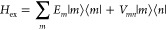
3contains the local excitation energies (site energies) *E*_*m*_ of the chromophores and the excitonic couplings *V*_*mn*_ between them. These quantities are taken at the equilibrium position of nuclei in the electronic ground state of the complex. The exciton-vibrational coupling Hamiltonian *H*_ex-vib_ takes into account the variation of the local excitation energies by the vibrations. A linear dependence is assumed on normal mode coordinates *q*_ξ_
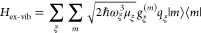
4with the vibrational frequency ω_ξ_, the reduced mass μ_ξ_, and the dimensionless coupling constant *g*_ξ_^(*m*)^ of normal mode ξ. The vibrational Hamiltonian *H*_vib_ is obtained from a normal-mode analysis of nuclear motion in the electronic ground state of the complex
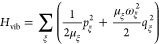
5Delocalized exciton states  are introduced as the eigenstates of *H*_ex_ with eigenenergies *E*_*M*_. In the exciton basis, the exciton-vibrational Hamiltonian *H*_ex-vib_ reads
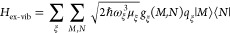
6with the dimensionless coupling constant
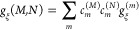
7We combine the diagonal part of *H*_ex-vib_ with *H*_ex_ and *H*_vib_ and introduce potential energy surfaces of the exciton states^52,53^
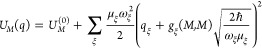
8where *q* comprises the vibrational coordinates, and the minimum of the PES is given as
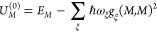
9The PESs of different exciton states are coupled by the off-diagonal elements of *H*_ex-vib_ in eq 6
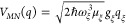
10with the off-diagonal coupling constant
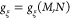
11(eq 7, with *M* ≠ *N*), which will be treated in perturbation theory below.

### Semiclassical Rate Constant in the Modified Redfield Theory

In the modified Redfield theory,^54−56^ it is assumed that nuclei, after optical excitation of the complex, relax in the PES of exciton states, defined above. After this nuclear equilibration, excitation energy transfer occurs between different PESs. We split the electronic Hamiltonian into a diagonal part *H*_0_ and a perturbation *V*
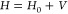
12where the diagonal part contains the PES *U*_*M*_ (eq 8) of exciton states
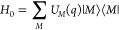
13and *V* comprises the couplings *V*_*MN*_ (eq 10) between different exciton states
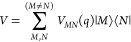
14The electronic wave function φ(*r*,*q*,*t*) of the complex is expanded with respect to the stationary states of *H*_0_
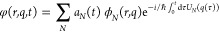
15where the time dependence of the electronic energy of state |*N*⟩ is given by the PES *U*_*N*_(*q*(τ)) and will be described by using a classical description of nuclear motion. Perturbation theory in the coupling *V* is used to obtain the coefficients *a*_*N*_(*t*) ≈ *a*_*N*_^(0)^(*t*) + *a*_*N*_^(1)^(*t*). It is assumed that the exciton is initially in state |*M*⟩. Hence, in zeroth-order in the inter-PES coupling we have
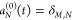
16From the electronic Schrödinger equation
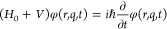
17taking into account the orthogonality of different excitonic states and the zeroth-order coefficient *a*_*N*_^(0)^ above, the time-derivative of the first-order coefficient is obtained as
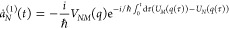
18The probability of finding the system, that was initially in state |*M*⟩, at time *t* in state |*N*⟩ is given as |*a*_*N*_^(1)^(*t*)|^2^, and the long-time limit of the transition probability per time, that is, the rate constant *k*_*M*→*N*_, follows as^57^
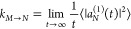
19where ⟨...⟩ denotes an average over thermally distributed initial coordinates and momenta of nuclei. With the integral of eq 18 and its complex conjugate, the rate constant becomes
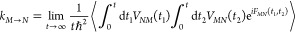
20where
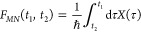
21with the energy gap between the final and the initial state
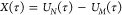
22Changing the integration variables in eq 20 to *t*_1_′ = *t*_1_ – *t*_2_ and *t*_2_ for *t*_1_ > *t*_2_ and to *t*_1_′ = *t*_2_ – *t*_1_ and *t*_2_ for *t*_1_ < *t*_2_, taking into account the Jacobian determinant |∂(*t*_1_′, *t*_2_)/∂(*t*_1_, *t*_2_)| = 1, gives
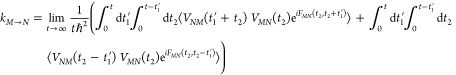
23The time dependence of the inter PES coupling *V*_*MN*_(*t*) and energy gap *X*(*t*) entering the function *F*_*MN*_(*t*_1_, *t*_2_) is determined by the equilibrium fluctuations of nuclei *q*(*t*) in the initial state |*M*⟩. In thermal equilibrium, the averages in the above integrals do not depend on the absolute time *t*_2_. Setting *t*_2_ = 0 in these expressions and using a substitution *t*_1_′ → −*t*_1_′ in the second line result in
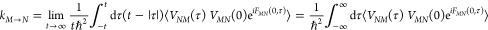
24The coupling *V*_*NM*_(τ) is given in eq 10, and the function *F*_*MN*_(0, τ) is obtained from eqs 21 and 22 and the PES of the two exciton states in eq 8, where we set the origin of the *q*_ξ_-axis to the minimum position of the PES *U*_*M*_(*q*) of the initial state |*M*⟩, as
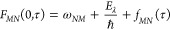
25with the transition frequency between the minima of the PES of the initial and the final exciton states |*M*⟩ and |*N*⟩, respectively, ω_*NM*_ = (*U*_*N*_^(0)^ – *U*_*M*_^(0)^)/*ℏ* and the reorganization energy *E*_λ_ that follows from the mutual displacement of the two PES
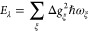
26containing the difference in diagonal exciton-vibrational coupling constants between the two states
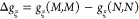
27The function *f*_*MN*_(τ) in eq 25 reads
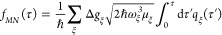
28A classical treatment of nuclear motion (*q̇*_ξ_ = ∂/∂*p*_ξ_*H*_vib_, *ṗ*_ξ_ = −∂/∂*q*_ξ_*H*_vib_ with the *H*_vib_ in eq 5) in the harmonic PES of the initial state |*M*⟩ (setting the origin of the *q*_ξ_ axis to the minimum position of *U*_*M*_(*q*)) results in
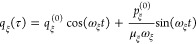
29with the initial values *q*_ξ_^(0)^ and *p*_ξ_^(0)^ of coordinates and momenta, respectively. Please note that this dynamics follows the Ehrenfest theorem, since it holds that *q̈*_ξ_ = −∂/∂*q*_ξ_⟨*M*|*H*_0_|*M*⟩ = −∂/∂*q*_ξ_*U*_*M*_(*q*), with the electronic Hamiltonian *H*_0_ in eq 13 and taking into account that the system is initially in exciton state |*M*⟩. The reorganization effects are described correctly, since only a single exciton state is excited initially.^43^

The rate constant *k*_*M*→*N*_ follows from eqs 24, 10, 25, and 28 as
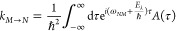
30where the function *A*(τ) reads
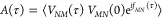
31with the *V*_*NM*_(τ) from eqs 10 and 29, and the *f*_*MN*_(τ) from eqs 28 and 29. The thermal average over initial coordinates and momenta in eq 31 is defined as
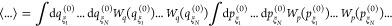
32with the Boltzmann factors
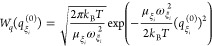
33and
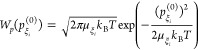
34Gaussian integrations result in
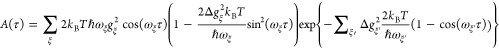
35By using a short-time approximation for the cosine function in the exponent, that is, , the function *A*(τ) can be written as
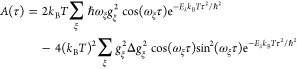
36with the reorganization energy of the diagonal parts of the exciton-vibrational coupling *E*_λ_ (eq 26). The second term on the right-hand side of eq 36 contains higher-order exciton-vibrational couplings resulting from the correlation between the diagonal (eq 27) and the off-diagonal (eq 7 with *M* ≠ *N*) contributions. Neglecting these correlations and performing the time-integration in eq 30 with the first term on the right-hand side of eq 36 result in the semiclassical modified Redfield rate constant
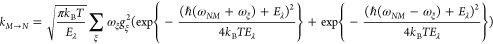
37which will be applied and analyzed in detail below.

## Semiclassical Rate Constant in the Generalized Förster Theory

Next, we turn to the generalized Förster theory^58−60^ that is used to describe exciton transfer between domains of pigments with strong intradomain and weak interdomain excitonic couplings. In this case, the exciton Hamiltonian is expanded in terms of the delocalized exciton states of the domains
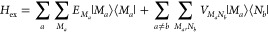
38with the coupling between exciton state |*M*_*a*_⟩ in domain *a* and |*N*_*b*_⟩ in domain *b*.
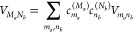
39where  is the excitonic coupling between pigment *m*_*a*_ in domain *a* and pigment *n*_*b*_ in domain *b*, and  and  are the coefficients of these two pigments in the respective exciton states |*M*_*a*_⟩ and |*N*_*b*_⟩. As before, the exciton-vibrational coupling is taken into account by introducing PES  of exciton states (eq 8), and the overall Hamiltonian (eq 12) is split into a part *H*_0_ and a perturbation *V*. *H*_0_ contains the PES of exciton states of the different domains *d*
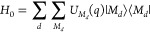
40and *V* the interdomain excitonic couplings
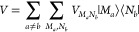
41A simplifying feature with respect to the semiclassical modified Redfield theory treated above is that  (eq 39) does not depend on the nuclear coordinates. Otherwise, the derivation of the rate constant goes along the same lines as above, and the rate constant  is obtained as
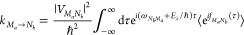
42with the energy difference  between the minima of the PES of the initial state |*M*_*a*_⟩ and that of the final state |*N*_*b*_⟩, the reorganization energy *E*_λ_ given by eq 26 with
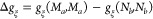
43and the function
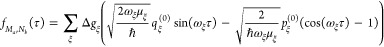
44Performing the average over the thermal distribution of initial coordinates and momenta yields
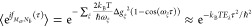
45where in the last step a short-time approximation for cos(ω_ξ_τ) was used and the reorganization energy *E*_λ_ in eqs 26 and 27, with *M* = *M*_*a*_ and *N* = *N*_*b*_, was introduced. Finally, the time integration in eq 42 results in the semiclassical Förster theory rate constant
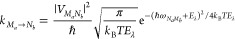
46which is formally identical to the Marcus theory rate constant of nonadiabatic electron transfer^41,42^ and was recently derived by considering the high-temperature limit of the quantum expression of this rate constant.^61^ It is notable that the semiclassical generalized Förster theory rate constant strictly fulfills the principle of detailed balance,  = .

In the limit of uncorrelated site energy fluctuations of the pigments, the reorganization energy *E*_λ_, in the present case, where the excited pigments contributing to the exciton states |*M*_*a*_⟩ and |*N*_*b*_⟩ are located in different domains, is given as the sum of the reorganization energies of the two exciton states  with  where the local reorganization energy  of the pigments is defined as
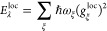
47We have assumed the same local coupling constant  for all pigments.

The rate constant  in eq 46 can then be expressed as
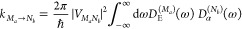
48containing the familiar overlap integral^42^ between the normalized line shape functions of the emissive transition *M*_*a*_ → 0 of the donor domain
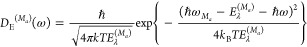
49with the 0–0 transition energy  of exciton state |*M*_*a*_⟩ and the absorptive transition 0 → *N*_*b*_ of the acceptor domain
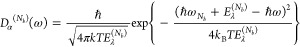
50The nuclear reorganization effects are seen as a Stokes shift  between the absorption maximum of the acceptor and the emission maximum of the donor, in case their 0–0 transition energies are equal ().

## Application

**Figure 1:**
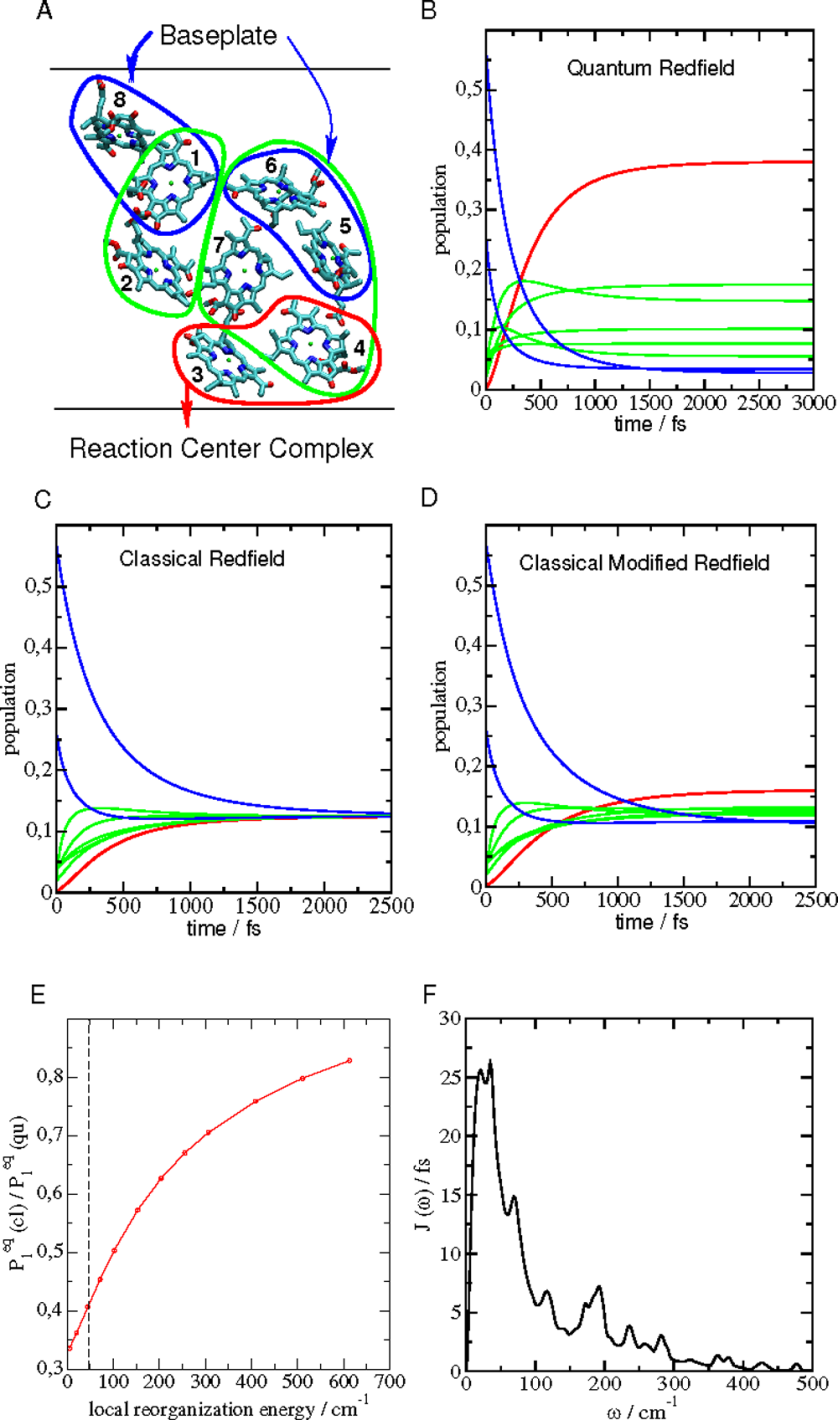
(A) Arrangement of BChl *a* pigments in the monomeric subunit of the FMO protein of *P. aestuarii*(62) in an orientation with the baseplate at the top and the reaction center complex at the bottom.^21,25,26^ Pigment contributing to high-, intermediate-, and low-energy exciton states are encircled in blue, green, and red, respectively. The blue and red arrows illustrate the flow of excitation energy. Graphics generated using VMD.^65^ (B) Population of high-, intermediate-, and low-energy exciton states after incoherent transfer from the baseplate, obtained from the Redfield theory using a quantum description of nuclear motion. (C) Same as in part B but using a classical description of nuclear motion. (D) Same as in parts B and C but using modified Redfield theory with a classical treatment of nuclear motion. (E) Ratio between equilibrium populations of the lowest exciton state obtained with semiclassical modified Redfield theory (*P*_1_^eq^(cl)) and a quantum description of nuclear motion (*P*_1_^eq^(qu)) as a function of the local reorganization energy *E*_λ_^loc^ (eq 47) of the pigments. The vertical dashed line marks the reorganization energy of the pigments in the FMO protein, obtained with the spectral density shown in panel F. For the other reorganization energies, the same functional form of *J*(ω) was assumed, and the amplitude was adjusted accordingly. (F) Local spectral density of the pigments *J*(ω) (eq 51) in the FMO protein, extracted from fluorescence line narrowing spectra and the temperature dependence of the linear absorption spectrum.^64^

**Figure 2:**
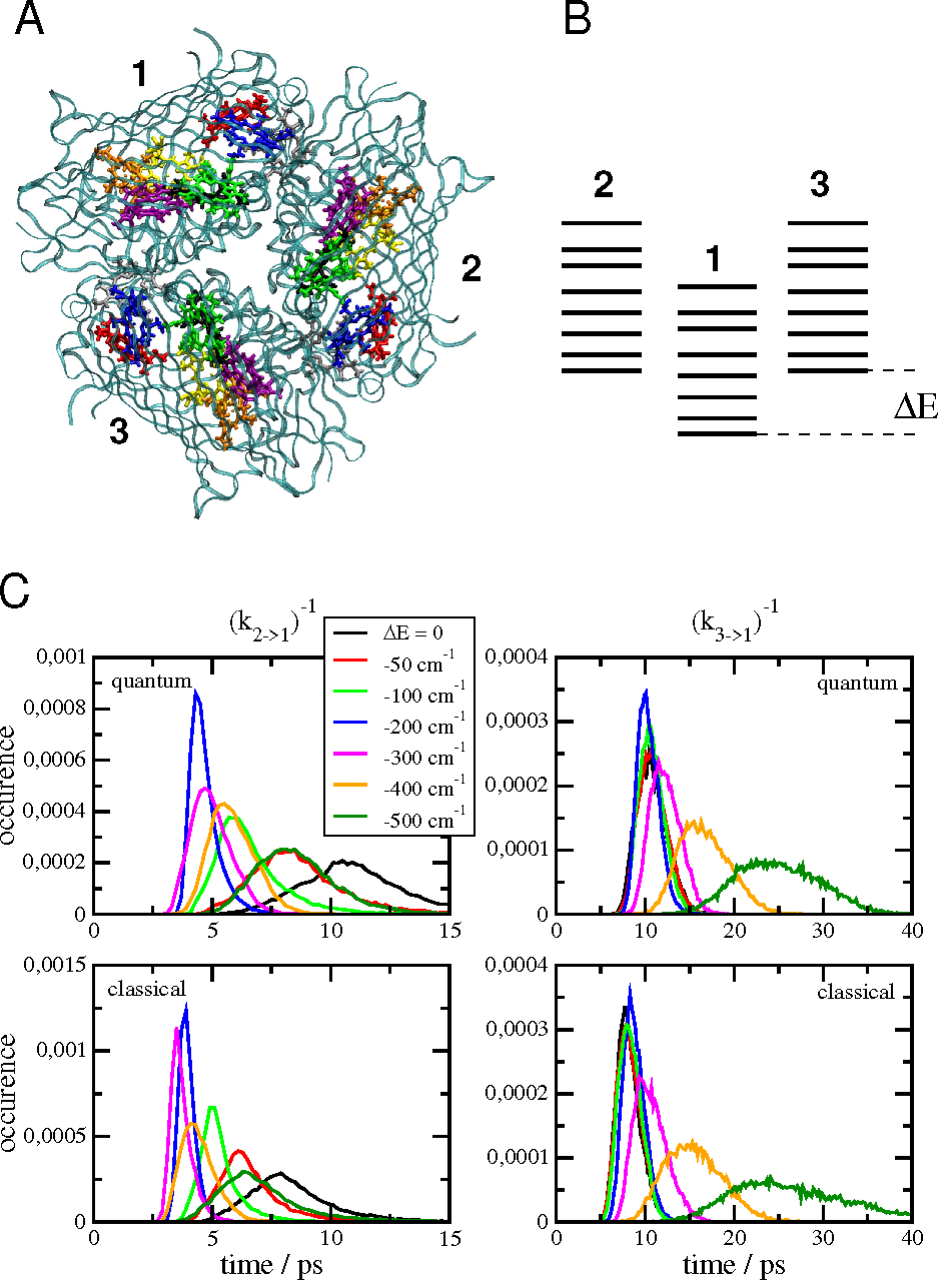
(A) Structure of the trimeric FMO protein of *P. aestuarii*(62) viewed normal to the membrane from the direction of the reaction center complex,^21,25,26^ protein shown in ribbon style, pigments shown in ball and sticks mode in different colors (BChl 1, red; BChl 2, blue; BChl 3, green; BChl4, purple; BChl 5, orange; BChl 6, yellow; BChl 7, black; BChl 8, gray). The phytyl tails of the pigments have been truncated for better visibility. Graphics generated with VMD.^65^ (B) Illustration of the exciton level structure of the three monomers. The energy levels of monomer 1 are downshifted by Δ*E* in the calculation of the intermonomer transfer times in panel C. (C) Histograms of inverse intermonomer rate constants *k*_2→1_^–1^ (left half) and *k*_3→1_^–1^ (right half) as a function of the energy difference Δ*E* between the monomers, illustrated in panel B. The quantum mechanical treatment of nuclear motion in the upper parts is compared with a classical treatment in the lower part.

We apply the present theories to the Fenna–Matthews–Olson (FMO) protein of green sulfur bacteria of *Prosthecochloris aestuarii*.^62^ The excitation energy collected by the outer chlorosome antenna enters the FMO protein via the baseplate and relaxes from the high-energy exciton states with contributions from the high-energy pigments via those with intermediate excitation energy to the low-energy exciton state with major contributions from BChls 3 and 4 located at the bottom of the complex (Figure 1A). Afterward, the excitation energy equilibrates between the three monomers of the trimeric complex (Figure 2A) and is transferred to the reaction center complex.^21^ The FMO protein has been an important model system for the development of experimental and theoretical techniques to study photosynthetic pigment–protein complexes, as reviewed recently.^28^ This review came to the conclusion that the most important nuclear quantum effect for the function of this complex is related to the dissipation of the excess energy of excitons. Here, we investigate, whether or not this conclusion also holds, if nuclear reorganization effects are taken into account, and considering also the intermonomer equilibration of excitation energy. Exciton relaxation in the monomeric subunit (Figure 1A) is described by the semiclassical modified Redfield theory (eq 37) and excitation energy transfer between the monomeric subunits (Figure 2A) by the semiclassical generalized Förster theory (eq 46). All calculations are performed assuming a physiological temperature (*T* = 300 K).

### Intramonomer Exciton Relaxation in the FMO Protein

We start by reproducing the earlier results^28^ obtained with the standard Redfield theory and its semiclassical variant, in which only the electronic motion is described quantum mechanically, whereas nuclei move according to Newton’s classical equations of motion. The initial population of exciton states was obtained by taking into account incoherent energy transfer from the baseplate. Please find the details of this description^63^ as well as the parameters (site energies, excitonic couplings, static disorder) in the Supporting Information of ref (28). The spectral density of the local exciton-vibrational coupling of the pigments
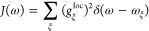
51was extracted from fluorescence line narrowing spectra and a fit of the temperature dependence of the absorption spectrum.^64^ It has a maximum around 30 cm^–1^ and an asymmetric shape extending up to 400 cm^–1^ (Figure 1F), resulting in a local reorganization energy *E*_λ_^loc^ (eq 47) of 45 cm^–1^. As expected, the quantum treatment of nuclear motion leads to a preferential population of low-energy exciton states (Figure 1B), whereas the semiclassical theory gives equal populations of all exciton states (Figure 1C) after exciton relaxation. The semiclassical modified Redfield theory, developed here (eq 37), provides a small preference to the equilibrium population of the low-energy exciton state (Figure 1D), but these populations are much closer to the classical Redfield limit than to the correct quantum result. Increasing the local reorganization energy significantly enhances the population of the lowest exciton state obtained in the semiclassical modified Redfield theory (Figure 1E). For *E*_λ_^loc^ = 500 cm^–1^, this population reaches 80% of the value resulting from the principle of detailed balance.

### Intermonomer Excitation Energy Transfer in the FMO Protein

Intermonomer excitation energy transfer in the FMO protein was recently shown to occur with a time constant of about 10 ps at room temperature, which is about 2 orders of magnitude slower than intramonomer exciton relaxation.^61^ Taking into account fast intramonomer exciton equilibration (assuming a correct thermalization), the rate constant between monomers *a* and *b* is obtained as
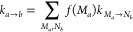
52with the Boltzmann factor *f*(*M*_*a*_) =  and the semiclassical generalized Förster theory rate constant  (eq 48). For a comparison, we also calculate the quantum rate constant that is obtained by replacing the Gaussian line shape functions for donor emission and acceptor absorption in eqs 49 and 50, respectively, by their quantum analogues^53,60^ that will be discussed in detail later.

Since both the semiclassical and the quantum generalized Förster theory rate constants fulfill the principle of detailed balance, we compare the time scale on which the equilibrium populations are obtained in the two theories. In order to obtain additional information on the performance of the semiclassical theory, we downshifted all exciton states of one of the three monomers (monomer 1) by a certain energy Δ*E* (Figure 2B) and studied the histogram of inverse rate constants *k*_2→1_^–1^ and *k*_3→1_^–1^ resulting from different realizations of static disorder in site energies of the pigments in dependence on Δ*E* (Figure 2C). The two transfer processes behave differently with respect to changes in Δ*E*. The transfer from monomer 2 to monomer 1 becomes faster for decreasing (more negative) Δ*E* between 0 and −200 (−300) cm^–1^ and slows down for Δ*E* = −400 and −500 cm^–1^, reaching similar time constants for Δ*E* = −50 cm^–1^ and Δ*E* = −500 cm^–1^. This behavior is reminiscent of the “normal”, “activationless”, and “inverted” regions of the Marcus theory of electron transfer, respectively.^41,42^ Qualitatively similar behavior is obtained for quantum and classical treatments of nuclear motion. The largest deviations occur for Δ*E* = 0, where the average classical time constant is about 25% smaller than the quantum value.

The transfer from monomer 3 to 1 is relative insensitive to lowering the energy of monomer 1 between Δ*E* = 0 and −200 cm^–1^ and significantly slows down from an average time constant of 10 ps at Δ*E* = −300 cm^–1^ to 15 and 25 ps at Δ*E* = −400 cm^–1^ and −500 cm^–1^, respectively. In this case, only the “activationless” and “inverted” regions of the reaction are observed. When the energy is lower, the distribution function of time constants becomes broader. The quantum behavior is almost quantitatively reproduced by the classical theory of nuclear motion.

## Discussion

In the limit of vanishing nuclear reorganization energy, *E*_λ_ → 0, the present semiclassical modified Redfield rate constant (eq 37) becomes equal to the semiclassical Redfield rate constant^28^
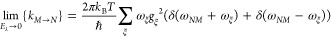
53In this case, equilibration leads to equal occupation probabilities of all exciton states, independent of their energies.

**Figure 3:**
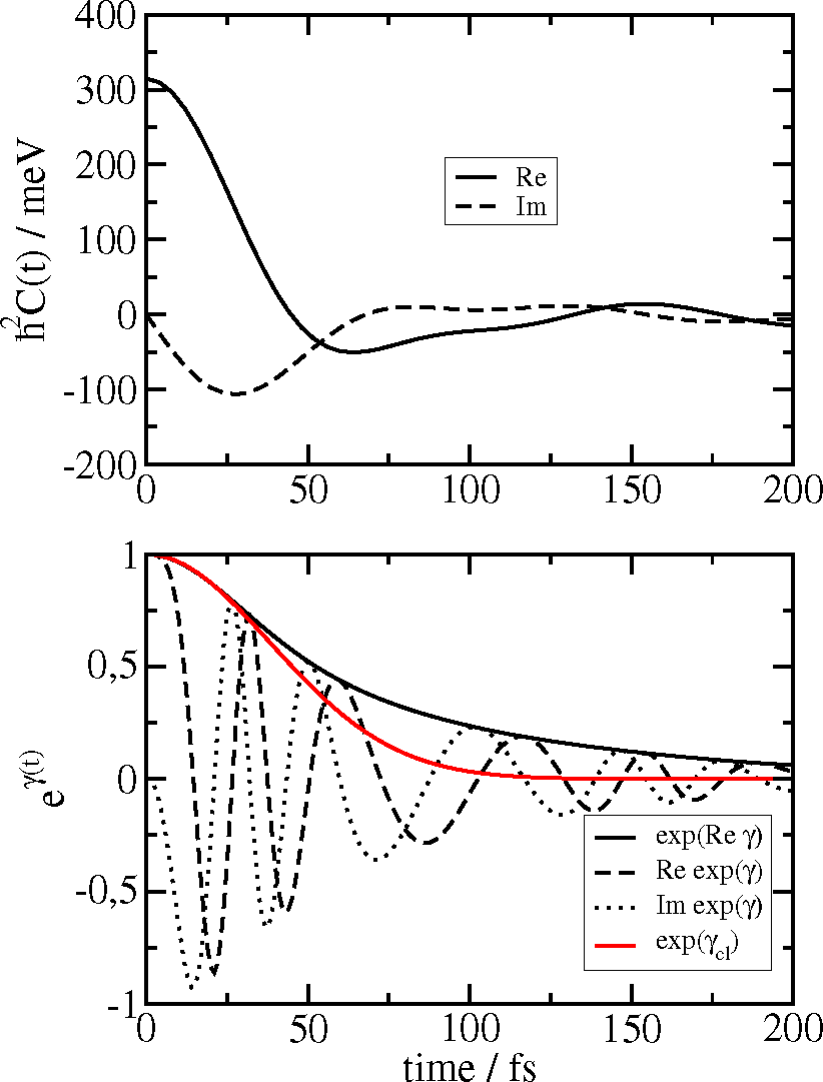
Upper part: real (solid line) and imaginary part of the correlation function *C*(*t*) (eq 54) of the local energy gap of the pigments obtained for the spectral density *J*(ω) in Figure 1F. Lower part: function e^γ(*t*)^ relevant for the calculation of optical line shapes, where γ(*t*) (eq 62) is related to the function *C*(*t*) in the upper part by eq 61. The short-time approximation  (red solid line), with the γ_cl_(*t*) in eq 63, leading to the semiclassical line shape function is compared to the quantum result that contains an imaginary (black dotted line) and a real (dashed dotted line) part. In addition, the amplitude e^Reγ(*t*)^ of the quantum result (solid black line) is shown.

In order to see, which particular aspect of classical nuclear motion gives rise to this defect, we examine the transition from the quantum Redfield rate constant to the above expression. In the quantum case, the Redfield rate constant is obtained as  with the Fourier transform  of the correlation function  where *V*_*NM*_ is the coupling between exciton states that depends linearly on the normal mode coordinates (eq 10); the time dependence is given by the *H*_vib_ in eq 5, and the ⟨...⟩ denotes an expectation value with respect to the equilibrium statistical operator of the vibrations *W*_eq_ =  exp(−*H*_vib_/*k*_B_*T*)/Tr{exp(−*H*_vib_/*k*_B_*T*)}. Please note that, in the Redfield theory, the mutual displacements between excitonic PESs are neglected; that is, no nuclear reorganization effects upon exciton relaxation are taken into account. Using eq 10 and introducing creation and annihilation operators of vibrational quanta of the ξth normal mode *C*_ξ_^†^ and *C*_ξ_, respectively, as  and , the correlation function becomes , with the correlation function *C*(*t*) of the local energy gap of the pigments
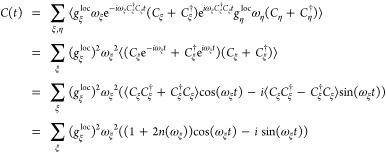
54The expectation value in front of the cosine term in the third line ⟨*C*_ξ_^†^*C*_ξ_ + *C*_ξ_*C*_ξ_^†^⟩ is proportional to ⟨*q*_ξ_^2^⟩ whereas that in front of the sine term in the fourth line ⟨*C*_ξ_*C*_ξ_^†^ – *C*_ξ_^†^*C*_ξ_⟩ is proportional to ⟨*q*_ξ_*p*_ξ_⟩. In a classical world, it holds that ⟨*q*_ξ_*p*_ξ_⟩ = ⟨*q*_ξ_⟩⟨*p*_ξ_⟩ = 0, whereas in a quantum world the uncertainty principle between coordinates and momenta leads to a correlation between the two that leaves ⟨*q*_ξ_*p*_ξ_⟩ and thereby the imagery part of *C*(*t*) nonzero. With the help of the commutator relation [*C*_ξ_, *C*_ξ_^†^] = 1, we obtain the last line of eq 54 containing the mean number of vibrational quanta that are excited at a given temperature *T* (Bose Einstein distribution function)
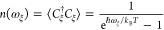
55The correlation function *C*(*t*) resulting for the present spectral density (Figure 1F) is shown in the upper part of Figure 3. The major part of the correlation function decays in less than 50 fs. The amplitude of the imaginary part of the correlation is smaller than that of the real part but clearly not zero.

The semiclassical Redfield theory rate constant in eq 53 follows by neglecting the imaginary part and by using a high-temperature approximation for the Bose–Einstein distribution function *n*(ω) ≈ *k*_B_*T*/(*ℏω*). The former approximation is responsible for the equal “uphill” and “downhill” rate constants. Without these approximations, the quantum Redfield rate constant is obtained
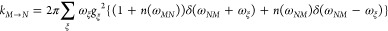
56which fulfils the principle of detailed balance. This result gave rise to our earlier conclusion that^28^ “..., in a world, where the behavior of electrons is governed by the fundamental equations of quantum mechanics and that of the nuclei by classical physics, there would be no preferential ‘downhill’ energy flow.”

The present semiclassical modified Redfield rate constant (eq 37) demonstrates that a classical treatment of nuclei can lead to a preferential downhill energy transfer, if *E*_λ_ ≠ 0. Additional insight can be obtained by expressing this *E*_λ_ (eq 26), using eqs 7 and 27, as
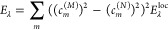
57with the reorganization energy *E*_λ_^loc^ (eq 47) of the local electronic transition of the chromophores, where we have neglected the correlations in site energy fluctuations between different pigments. This approximation is justified for pigment–protein complexes by a normal-mode analysis of the spectral density of the FMO protein.^66,67^ For completely delocalized excited states, the probability (*c*_*m*_^(*M*)^)^2^ of finding chromophore *m* excited is equal for all exciton states. Therefore, in this limit the reorganization energy *E*_λ_ vanishes, and the modified Redfield rate constant in eq 37 becomes the Redfield rate constant in eq 53 with the consequence that there is no preferential downhill energy transfer. This result is consistent with the finding of Ishizaki and Fleming that a neglect of the local nuclear reorganization effects leads to equal equilibrium excited state populations of the pigments in an Ehrenfest-type semiclassical treatment.^43^

If there is partial localization of excited states, e.g., by different local transition energies of the chromophores, *E*_λ_ ≠ 0, and it holds that *k*_*M*→*N*_/*k*_*N*→*M*_ > 1 for *ℏ*_*MN*_ > 0. Therefore, the equilibrium population of low-energy exciton states is higher than that of the high-energy states. If the nuclear reorganization energy *E*_λ_ is large compared to the vibrational quanta *ℏω*_ξ_ of the exciton-vibrational coupling, the semiclassical modified Redfield rate constant in eq 37 becomes
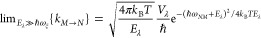
58where the reorganization energy *V*_λ_ of the off-diagonal exciton vibrational coupling (eq 7 with *M* ≠ *N*) was introduced as . In this limit, the rate constant fulfills the principle of detailed balance (eq 1), despite the classical treatment of nuclear motion.

However, in photosynthetic pigment–protein complexes, *ℏω*_ξ_ is in the same range as *E*_λ_. A typical example for the spectral density J(ω) of these complexes is shown in Figure 1F. J(ω) extends up to vibrational energies of 400–500 cm^–1^ and results in a local reorganization energy *E*_λ_^loc^ of only 45 cm^–1^. Consequently, the equilibrium populations obtained with the semiclassical modified Redfield theory (Figure 1D) are much closer to the equal populations obtained in the classical Redfield theory (Figure 1C) than to the realistic values of the quantum Redfield theory (Figure 1A). In order to reach 80% of the quantum population of the lowest exciton state with the classical theory of nuclear motion, the reorganization energy would have to be increased by an order of magnitude (Figure 1E). Such high reorganization energies are not observed for photosynthetic light-harvesting complexes. Hence, we conclude that the uncertainty principle between nuclear coordinates and momenta is essential for the preferential population of low-energy exciton states of strongly coupled pigments in photosynthetic antennae. The reorganization of nuclei during exciton relaxation that can be captured by a classical theory of nuclear motion is not strong enough to have a significant influence on the equilibrium population of excitons.

This situation changes if the excitonic couplings between pigments become weaker, a situation encountered in all photosynthetic complexes at some length scale, where a nonhomogeneous distribution of pigments, bound to the same or to different subcomplexes, is observed like, e.g., in the photosynthetic supercomplexes of plants and algae.^67^ In this case, excitons delocalize in certain domains of strongly coupled pigments, and interdomain exciton transfer occurs incoherently. In this case, both the quantum and the classical treatment of unclear motion lead to the correct equilibrium population of exciton states.

In order to understand why in this case the uncertainty between nuclear momenta and coordinates is less important, we start by discussing the quantum expression for the rate constant in the generalized Förster theory. We have to replace the semiclassical emission and absorption line shape functions in eqs 49 and 50 by their quantum mechanical counterparts reading
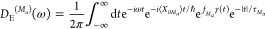
59and
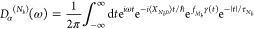
60respectively, where  and  denote the energy gap between the final and the initial state of the optical transition. Please note that, in eqs 59 and 60, the off-diagonal elements of the exciton-vibrational coupling (eq 6 with *M* ≠ *N*) are treated in secular approximation^53,61^ and lead to the exciton relaxation induced dephasing times  and , which are determined by the Redfield rate constants ,^53^. The diagonal elements were treated here with a second-order cumulant expansion^42,68^ that is exact for the present harmonic PES and relates the line shape function to the energy gap correlation function of the pigments *C*(*t*) (eq 54).

The expectation value ⟨...⟩ has to be taken with respect to the equilibrium statistical operator of the vibrational degrees of freedom of the initial state, , and γ(*t*) is given as
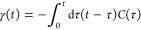
61with the correlation function *C*(τ) in eq 54. The origin of the coordinate axis is conveniently put at the minimum position of the PES of the initial state, and the expectation values of the energy gap to the final state with a shifted PES are obtained as  and .

As we learned before, the imaginary part of the correlation function *C*(*t*) (eq 54) originates from the quantum mechanical uncertainty principle between nuclear coordinates and momenta. Integration according to eq 61 results in
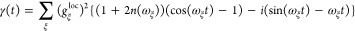
62Interestingly, the imaginary part of γ(*t*), which follows from the imaginary part of *C*(*t*), vanishes for short times. Hence, for a fast decay of the energy gap correlation function of the pigments, the uncertainty principle is not as critical in the generalized Förster as in the Redfield theory. In this limit, a short time approximation [cos(ω_ξ_*t*) ≈ 1 – ω_ξ_^2^*t*^2^/2, sin(ω_ξ_*t*) ≈ ω_ξ_*t*] together with a high-temperature approximation *n*(ω_ξ_) ≈ *k*_B_*T*/*ℏω*_ξ_ gives
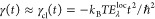
63that we have denoted as γ_cl_(*t*), since with eqs 59 and 60, neglecting the lifetime broadening  and , the semiclassical line shape expressions in eqs 49 and 50 result.

A comparison between the full exp(*γt*) and its short-time approximation γ_cl_(*t*) leading to the semiclassical line shape function is shown in the lower half of Figure 3. The function exp(γ_cl_(*t*)) decays on a similar time scale as the full exp(*γt*). The latter, however, due to the *i* sin(ω_ξ_*t*) contributions, is complex and contains oscillations that are absent in the real short-time approximation. These oscillations describe vibrational sidebands that are only roughly included in the broadening of the semiclassical Gaussian line shape function [by the somewhat faster decay of the exp(γ_cl_(*t*))]. Interestingly, the semiclassical generalized Förster theory provides a qualitatively correct description of the interdomain exciton transfer including relatively large energy differences (Figure 2C). For even larger energy gaps, as they occur between chemically distinct pigments, the thermal broadening of the symmetric semiclassical Gaussian line shape function will not be enough, and additional high-frequency intramolecular vibrations of the pigments have to be taken into account. This inclusion can only be done in a quantum theory since the vibrational sidebands of these high-frequency vibrations will occur only on the high-/low-energy sides of the 0–0 transition in absorption/emission. A classical theory of nuclear motion cannot describe this asymmetry.^40^ Again, it is the missing imaginary part of the correlation function resulting from the uncertainty principle between nuclear coordinates and momenta that is responsible for this defect of the semiclassical line shape theory. An approximate inclusion of these effects can be obtained by treating a single effective vibrational mode quantum mechanically and the remaining modes classically.^42,69,70^

Large energy gaps between chromophores occur, e.g., between chlorophyll *b* (Chl *b*) and Chl *a* pigments in the major light-harvesting complex of higher plants LHCII, where high-frequency intrapigment vibrations were found to be essential for efficient Chl *b* → Chl *a* energy transfer.^71^ High-frequency intrapigment vibrations were also found to be important for energy transfer between high- and low-energy bilin chromophores in marine cryptophyte algae.^72−74^ In these cases, where the energy difference between the minima of the PES of the excited states is large compared to the reorganization energy, a classical theory of nuclear motion would result in a too small rate constant. Larger reorganization energies occur in charge transfer reactions, because of the polar nature of the charge separated state. Despite a very small effect of single high-frequency intrapigment vibrations, collectively these modes were found to decrease the rate constant in the normal region and to increase it in the inverted region of primary electron transfer in photosystem II, making the reaction robust against static disorder effects.^75^

## Conclusions

We have investigated exciton relaxation in domains of strongly coupled pigments and exciton transfer between such domains with weak interdomain excitonic couplings, using the semiclassical modified Redfield and generalized Förster theory, respectively. A key quantity appears to be the energy gap correlation function of these reactions. Whereas a classical theory of nuclear motion results in a real correlation function, the uncertainty principle between nuclear coordinates and momenta in a quantum theory gives rise to an imaginary part. This imaginary part is found responsible for the equilibration of excitation energy in domains of strongly coupled pigments. Nuclear reorganization effects also lead to a preferential population of low-energy exciton states, but for realistic spectral densities of photosynthetic light-harvesting antennae, these reorganization effects are much too small to compensate for the missing uncertainty principle in a classical theory of nuclear motion.

The situation changes for weaker excitonic couplings as they occur between different exciton domains in photosynthetic antennae. In this case, the nuclear reorganization effects guarantee a correct thermal equilibration of excitation energy, independent of the correlations between nuclear coordinates and momenta. The uncertainty principle still has an influence on the time scale of the reaction, in particular if large energy gaps are involved.
